# Ethnomedicinal, Chemical, and Biological Aspects of *Lannea* Species—A Review

**DOI:** 10.3390/plants13050690

**Published:** 2024-02-29

**Authors:** Quintino Malú, Gonçalo I. Caldeira, Luís Catarino, Bucar Indjai, Isabel Moreira da Silva, Beatriz Lima, Olga Silva

**Affiliations:** 1 Research Institute for Medicines (iMed.ULisboa), Faculty of Pharmacy, Universidade de Lisboa, 1649-003 Lisbon, Portugal; quintinomalu@campus.ul.pt (Q.M.); goncalo.caldeira@campus.ul.pt (G.I.C.); isabelsilva@edu.ulisboa.pt (I.M.d.S.); mblima@ff.ulisboa.pt (B.L.); 2 Centro de Ecologia, Evolução e Alterações Ambientais, (cE3c) & CHANGE-Global Change and Sustainability Institute, Faculdade de Ciências, Universidade de Lisboa, 1749-016 Lisboa, Portugal; lmcatarino@gmail.com; 3 Instituto Nacional de Estudos e Pesquisa, Avenida dos Combatentes da Liberdade da Pátria, Bissau 112, Guinea-Bissau; inddjai.b@gmail.com

**Keywords:** *Anacardiaceae*, biological activity, ethnomedicine, *Lannea*, inflammation

## Abstract

*Lannea* L. genus belongs to the *Anacardiaceae* botanical family and has long been used in traditional medicinal systems of many countries to manage several health conditions, but no studies have been conducted regarding its usefulness as a source of herbal medicine for human use. A literature review was conducted on scientific papers indexed on B-On, Pubmed, and Web of Science databases. Our results showed that medicinal plants from this botanical genus, mostly constituted by bark and leaf, are often used to approach a wide variety of disease symptoms, like fever, inflammatory states, pain, and gastrointestinal disorders. Phytochemical profiles of *Lannea* species revealed that phenolic acid derivatives including hydroquinones, phenolic acids, flavonoids, condensed tannins, and triterpenoids are the main classes of secondary metabolites present. Among the total of 165 identified compounds, 57 (34.5%) are flavonoids, mostly quercetin- and myricetin-derived flavonols and catechin and epicatechin flavan-3-ol derivatives also containing a galloyl group. In vitro and in vivo studies allowed the identification of 12 different biological activities, amongst which antimicrobial, antioxidant, anti-inflammatory, and cytotoxic activities were the most frequently cited and observed in in vitro essays. Our review contributes useful information for the scientifical validation of the use of *Lannea* species in traditional medicinal systems and shows that more research needs to be conducted to better understand the concrete utility of these as herbal medicines.

## 1. Introduction

*Lannea* A. Rich. in Guill. is an important genus of flowering plants in *Anacardiaceae*, a botanical family comprising 81 genera and 800 species spread across tropical and subtropical regions with warm or temperate climates (tropical and South Africa, Saudi Arabian Peninsula, India, China, and Indochina) [1]. In addition to their importance in traditional medicinal systems, some species of *Anacardiaceae* have high economic value (e.g., *Anacardium occidentale* L., *Mangifera indica* L.) due to the use of their fruits and seeds in food and manufacture of beverages, being important to local communities and national economies as renewable forest resources and tradeable products [2,3]. The ancestral knowledge of Traditional Medicine Practitioners about the different medicinal proprieties of distinct species of this family has benefited many communities of tropical and sub-tropical countries where access to conventional primary health services is scarce and also in more developed countries as a complementary use to conventional medicine [4].

The *Lannea* genus was first described by Achille Richard and published in Florae Senegambiae Tentamen 153 in 1831 [5]. According to The World Flora Online [6], it includes a total of 36 accepted species (Table 1), of which 14.2% are classified as data deficient (DD), 30.9% as least concern (LC), 4.7% are classified as least concern (NT), and 7.1% as vulnerable (VU). 

*Lannea* species are mainly trees, shrubs, or dioecious subshrubs up to 15 m high and are known for their great morphological diversity, distributed in the tropical and subtropical zones and native to tropical Africa and Asia. These species have characteristic imparipinnate leaves, opposite, entire leaflets, and a terminal panicle or raceme as the inflorescence. Most of them are deciduous and can be found in humid, arid, and dry environments but not in deserts or at altitudes over 3500 m [4,8]. 

To exemplify the botanical characteristics of *Lannea* species, we hereby provide a description of various species within the genus, including *L. coromandelica*, *L. velutina*, *L. schimperii*, *L. acida*, *L. microcarpa*, and *L. welwitschii*, focusing on their leaf morphology and anatomical characteristics. The leaves of *Lannea* species are compound and imparipinnate, consisting of petiolate leaflets that are oppositely arranged, forming a pseudo-verticillate pattern in the case of *L. coromandelica*. The leaves exhibit membranous texture, oval shape with asymmetric bases and pointed apices, and entire margins. Leaf size varies, and primary venation is pinnate. Secondary venation displays weak brochidodromous patterns with six basal veins, and intersecondary veins are faint. Tertiary venation demonstrates a mixed arrangement (opposite/alternate), while fourth-order venation is regularly polygonal reticulate. Fifth-order veins are dichotomous, and the highest order observed is the sixth. Marginal venation is free, forming incomplete arches. Stomata of the policytic-anomocytic type are located exclusively on the abaxial surface. Trichomes, which are moderate, multicellular, and stellate, are distributed throughout the leaf surface, and no prismatic crystals, druses, or resinous canals are observed. Regarding *L. schimperii*, *L. acida*, *L. microcarpa*, and *L. welwitschii*, these species exhibit several anatomical and morphological characteristics typical of the genus, despite the existing variability within and between some genera. Straight, curved, round, and wavy cell walls, as well as polygonal shapes, are observed on both leaf surfaces. Stomata are confined to the abaxial surface in all species, with only cyclocytic and anomocytic types identified. Trichomes are present in some *Lannea* species, with *L. schimperii* being the only one possessing trichomes on both adaxial and abaxial surfaces [9,10,11].

Modern medicine and scientific developments contribute to creating better health conditions in industrialized countries through constant breakthroughs in many areas. However, the global demographic distribution shows us that most of the world’s population lives in countries that do not have access to such healthcare. In these countries, people still rely almost exclusively on traditional medical systems, whose practices are based on the use of medicinal plants to treat illness or promote healthy conditions. Research shows that the ethnobotanical uses of *Lannea* species are well recognized in countries where they are native and includes their use as medicine, food, and ornamental and domestic lumber [12,13]. 

The use of *Lannea* species as medicinal plants in traditional medicinal systems is widely accepted, but there is a need for a critical assessment of their potential as a source of effective medicines based on quality, effectiveness, and safety data. A literature review of the available scientific information on *Lannea* species regarding their ethnomedical uses as well as their chemical, pharmacological, and toxicological data are hereby presented. This work is expected to provide a deep understanding of the potential of this botanical genus as a source of effective medicinal plants.

## 2. Results

### 2.1. Selection of Information

**Figure 1:**
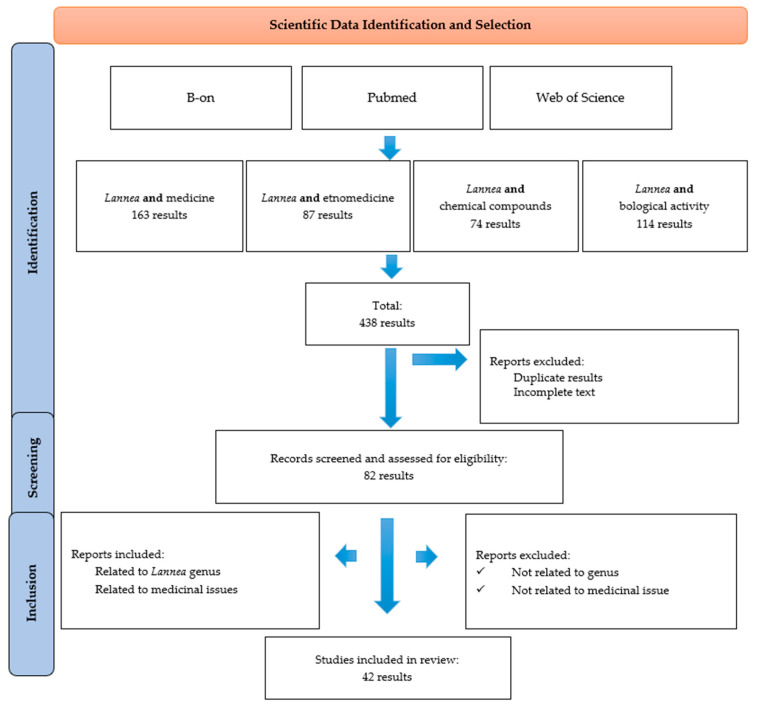
Data screening based on the PRISMA methodology.

Data collection and selection were made according to the scheme presented in Figure 1. Initially, the database search of the scientific literature yielded 438 results. After excluding duplicate results, 82 scientific reports were assessed for relevance. Next, irrelevant reports were eliminated, and finally, 42 scientific publications were considered eligible for detailed analysis.

### 2.2. Ethnobotanical and Ethnomedical Data

#### 2.2.1. Vernacular Names

The genus *Lannea* includes 16 species used in traditional medicinal systems (41.6% of the total number of accepted *Lannea* species) that are distributed in several countries, most of them in the African continent. Table 2 shows the vernacular names of the various species used in these traditional medicinal systems.

#### 2.2.2. Traditional Uses

Table 3 summarises the obtained data on the traditional medicinal uses of the 14 *Lannea* species. Results showed that for most of them there is little information about the exact methodology and duration of treatment. Bark (29%) and leaf (17%) were the most used plant parts, and the most reported symptoms and illnesses were related to infection symptoms (31%), gastrointestinal discomfort (14%), pain (12%), diarrhoea (9%), and inflammation (7%). *Lannea coromandelica* and *Lannea edulis* are the most reported species and are used in traditional medicine systems of 15 and 14 countries, respectively. 

Other *Lannea* species, like *L. acida*, are employed in tropical Africa to treat and manage bacterial, fungal, and viral infections, fever, and mental and gastrointestinal disorders. For example, *L. acida* is used to treat dysentery, stomach pain, and other gastrointestinal pathologies [29]; *L. microcarpa* is used for the treatment of mouth blisters, rheumatism, dysentery, diarrhoea, gastroenteritis, malaria, and bacterial infections [30]; *L. schweinfurthii* is used for the treatment of diseases related to the reproductive system, circulatory system, and gastrointestinal diseases, headaches, and against opportunistic diseases related to HIV, such as malaria, diarrhoea, tuberculosis, and skin infections [31].

*Lannea ambacensis* is known to be used in traditional Angolan medicine, particularly in the treatment of diabetes, rheumatism, and symptoms of respiratory, gastrointestinal, and urogenital diseases [32].

**Table 3:** Traditional uses of *Lannea* species by geographical region.

Species	Distribution	Medicinal Uses	Plant Part
*Lannea acida* [33]	Benin, Burkina Faso, Ghana, Guinea-Bissau, Ivory Coast, Niger, Nigeria, Senegal, Togo	Antipyretic, gastrointestinal tract disorder, malaria, pain, skin disease, and sexually transmitted disease (gonorrhoea, syphilis)	Branch, root, stem, stem bark
*Lannea alata* [15]	Kenya, Somalia, South Africa, Tanzania	Fever, fractures, malaria	Stem
*Lannea ambacensis* [16]	Angola	Asthma, colitis, cough, eye diseases, ulcer	Root
*Lannea angolensis* [17]	Angola	Bronchitis, pleuropneumonia, pneumonia, rhinitis, tuberculosis	Bark
*Lannea barteri* [34]	Benin, Burkina Faso, Burundi, Democratic Republic of the Congo, Ethiopia, Ghana, Guinea-Conakry, Ivory Coast, Mali, Nigeria, Uganda, Zaire	Anaemia, convulsions, diabetes, oedema, epilepsy, leprosy, madness, paralysis, salmonellosis, spasms, vermifuge	Bark, leaf Stem bark
*Lannea coromandelica* [19,20]	Andaman, Assa, Bangladesh, Cambodia, Guangdong, Guangxi Hainan, India, Laos, Myanmar, Nepal, Sri Lanka, Thailand, Vietnam, Yunnan	Heart disease, inflammations, leprous ulcers, mouth sores, pain, rashes, sprains, toothache	Bark, leaf
*Lannea edulis* [35]	Angola, Botswana, Burundi, Democratic Republic of the Congo, Ethiopia, Kenya, Malawi, Mozambique, Rwanda, South Africa, Tanzania, Uganda, Zambia, Zimbabwe	Bilhárzia and other parasitoses, cholera, contusion, diarrhoea, fever, food, haematoma, malaria, sexually transmitted disease (gonorrhoea, syphilis), swelling, tuberculosis, wound	Fruit, leaf, root, root bark, stem
*Lannea humilis* [23]	Ethiopia, Senegal, Zambia, Zimbabwe	Body aches, cholera, cough, diarrhoea, dysentery, nausea, weakness	Bark
*Lannea nigritana* [24]	Benin, Cameroon, Central African Republic, Congo (Brazzaville), Equatorial Guinea, Gambia, Ivory Coast, Liberia, Mali, Nigeria, Senegal, Sierra Leone, Togo	Anaemia, bad odour, cachexia, chest stiffness, drepanocytosis, dysentery, impotence, intestinal pain, purgative, rickets, tiredness	Bark
*Lannea rivae* [36]	Ethiopia, Kenya, Tanzania, Uganda	Cold, cough, stomach-ache	Bark
*Lannea schimperi* [37]	Burundi, Cameroon, Congo, Ethiopia, Kenya, Malawi, Mozambique, Nigeria, Rwanda, Tanzania, Togo, Uganda, Zambia	Back pain and general weakness, diarrhoea, dysentery, infections, stomach pain, tuberculosis	Bark, branch, leaf, stem, trunk
*Lannea schweinfurthii* [38]	Botswana, Ethiopia, Kenya, Malawi, Mozambique, Rwanda, Somalia, Sudan, Tanzania, Uganda, Zambia, Zimbabwe	Abdominal pain, anaemia, diarrhoea, food, gastric ulcer, headaches, sexually transmitted diseases (chlamydia, gonorrhoea, syphilis), stomach problems, tonic	Bark, leaf, stem bark
*Lannea velutina* [27]	Burkina Faso, Central African Republic, Chad, Ghana, Guinea-Bissau, Senegal	Anaemia, asthenia, cachexia, cholera, conjunctivitis, cuts, diarrhoea, dysentery, ectoparasites (flea, leech, lice, mite, tick), fever, impotence, inflammation, pain, rash, renal colic, skin growths, tuberculosis, wound	Bark, fruit, leaf, root
*Lannea welwitschii* [39]	Angola, Cameroon, Central African Republic, Congo, Ethiopia, Gabon, Ghana, Ivory Coast, Liberia, Nigeria, Uganda, Zaïre	Diarrhoea, dysentery, oedema, epilepsy, food, gout, haemorrhoids, hypertension, laxative, nasopharyngeal disorders, pulmonary diseases, purgative, venereal diseases	Bark, root

### 2.3. Phytochemical Studies

The results of the chemical studies conducted on *Lannea* species are summarised in Table 4. Most studies focused on leaf and bark plant parts. Polyphenolic compounds, including hydroquinones, phenolic acids, flavonoids, and terpenoids, namely triterpenoids, are the major classes of compounds identified in this botanical genus. Other terpenoid derivatives and fatty acids were also commonly identified. 

Among the total 160 compounds identified in *Lannea* species, 57 (34.5%) are flavonoids (quercetin and myricetin flavonols) and condensed tannins like catechin and epicatechin, also containing a galloyl group. As in other *Anacardiaceae* species, proanthocyanidins are representative secondary metabolites found in all parts of the plant, mainly in the bark. 

**Table 4:** Secondary metabolites of *Lannea* species.

Species, Ref.	Plant Part	Chemical Class	Compound
*L. acida* [40,41,42,43,44,45]	Whole plant	Flavonol	Quercetin
	Leaf	Flavanone	6,7-(2″,2″ -dimethyl chromene)-8-γ, γ-dimethyl allyl flavanone
		Flavonol	3′,4′dihydroxy-7,8(2″,2″-dimethyl chromene)-6-γ, γ dimethyl allyl flavonol
		Isoflavone	7-methyltectorigenin
		Isoflavone	Irisolidone
		Flavonol glycoside	Myricetin-3-O-α-L-rhamnopyranoside
		Flavonol glycoside	Myricetin-3-O-β-D-glucopyranoside
		Flavonol glycoside	Myricetin-3-(6″-galloylgalactoside)
		Gallic acid derivative	3,4,5-trigalloylquinic acid
		Flavan-3-ol	(-)-Epicatechin-3-gallate
		Flavan-3-ol	(-)-Epigallocatechin-3-gallate
		Flavan-3-ol	(-)-Epigallocatechin
		Flavan-3-ol	(-)-Epicatechin
		Flavone	Lanceolatin B
		Flavanone	7,2′-dimethoxy-4′,5′methylenedioxyflavanone
		Eugenol derivative	Eugenyl-O-β-D-(6′-sulphonylglucoside)
		Flavonol glycoside	Quercetin-3-O-β-D-glucuronic acid
		Flavonol glycoside	Quercetin-3-O-β-D-glucopyranoside
		Flavonol glycoside	Quercetin-3-(6″-galloylglucopyranoside)
	Stem bark	Flavone	Luteolin
		Flavonol	Kaempferol
		Fatty acid	Hexadecanoic acid (20.59%)
		Fatty acid	Trans-13-octadecenoic acid decanoic acid (2.16%).
		Fatty acid	7,10-octadecanoyl acid
		Fatty acid	Hexadecanoic acid
		Fatty acid	Ecadienoic acid
		Fatty acid	Eicosanoic acid (7.62%)
		Fatty acid	Dodecanoic acid (8.51%)
		Fatty acid	Octadecanoic acid (13.77%)
		Fatty acid	Tetradecanoic acid (18.18%)
		Methyl ester	Methyl ester (4.86%)
		Methyl ester	Methyl ester (7.70%)
		Ester	Methoxy acetic acid, 2-tetradecyl ester
		Phthalate ester	Dibutyl phthalate (4.12%)
	Root bark	Phenol derivative	(E)-3-(hepatic-14-enyl)phenol
		Phenol derivative	(E)-3-(nonadec-16-enyl)phenol
		Benzene derivative	(E)-2-(heptadec-14-enyl)benzene-1,4-diol
		Cyclohexenone	(5R,14E)-5-(heptadec-14-enyl)-5-hydroxycyclohex-2-en-1-one
		Cyclohexenone	(5R,16E)-5-(nonadec-16-enyl)-5-hydroxycyclohex-2-en-1-one
		Cyclohexene diol	(1S,3S)-1-((E)-heptadec-14-enyl)cyclohex-4-ene-1,3-diol
		Cyclohexene diol	(1S,3S)-1-((E)-nonadec-16-enyl)cyclohex-4-ene-1,3-diol
		Cyclohexene diol	(1S,3S)-1-((E)-heneicos-18-enyl)cyclohex-4-ene-1,3-diol
		Bicyclic alcohol	(1S,3S,6R)-1-((E)-heptadec-14-enyl)-7-oxabicyclo [4.1.0]hept-4-en-3-ol
		Bicyclic alcohol	(1R,3R,6S)-1-((E)-nonadec-16-enyl)-7-oxabicyclo[4.1.0]hept-4-en-3-ol
		Cyclohexenone	(4R,5S)-5-((E)-heptadec-14-en-1-yl)-4,5-dihydroxy-cyclohex-2-en-1-one
*L. alata* [46,47]	Whole plant	Flavonol	Lanneaflavonol
		Flavonol	Dihydrolanneaflavonol
		Flavonol glycoside	Myricetin-3-O-α ramnopyranoside
		Flavonol glycoside	Myricetin-3-O-α-arabinofuranoside (betmidin)
		Triterpene	Lupeol
		Phytosterol	ß-sitosterol
*L. barteri* [48]	Leaf	Flavonol glycoside	Kaempferol-3-O-rhamnoside
		Flavonol glycoside	Myricetin-3-O-rhamnoside
		Flavonol	Quercetin-3,7,3′,4′-tetramethyl
		Flavonol glycoside	Quercetin-3- O-arabinofuranoside
		Flavonol glycoside	Quercetin-3-O-galactoside (hysperoside)
		Flavonol glycoside	Quercetin-3-O-rhamnoside (quercetrin)
*L. coromandelica* [49,50,51]	Bark	Lipid derivative	(2S,3S,4R,10E)-2-[(2R)-2-hydroxytetracosanoyl amino]-10-octadecene-1,3,4-triol
		Phenolic aldehyde	Isovanillin
		Glycosphingolipid	Aralia cerebroside
		Saturated fatty acid	Palmitic acid
		Saturated fatty acid	Stearic acid
		Phenolic acid	Protocatechuic acid
		Oestrogenic compound	P-hydroxybenzoic acid ethyl ester
		Organic compound	5,5-dibuthoxy-2,2-bifuran
		Phytosterol ester	Phytosterol-β-sitosterol palmitate
		Sterol glycoside	Β-sitosteryl-3β-glucopyranoside-6-O-palmitate
		Triterpene	Myricadiol
	Leaf	Flavonol	Quercetin
		Flavonol glycoside	Quercetin-3-arabinoside
		Flavan-3-ol	Leucocyanidin
		Flavan-3-ol	Leucodelphinidin
	Flower, stem bark	Phytosterol	ß-Sitosterol
		Flavonol glycoside	Isoquercetin
		Flavonol	(2R, 3S)-(+)-4,7-di-O-methylhydroquercetin
		Flavonol	(2R, 3S)-(+))-4-O-methyldihydroquercetin
		Flavonol	(2R, 3S)-(+) 3,5-dihydroxy-4,7dimethoxydihydroflavonol
		Flavonol	(2R, 3S)-(+)-4,5,7-trimethoxydihydroflavonol
		Flavonol	(2R, 3S)-(+)-4,7-di-O-methyldihydrokaemferol
		Flavonol	Morin
		Oligosaccharide	4-O-(α-D-galactopyranosyluronic acid)-D-galactose
		Oligosaccharide	6-O-(ß-D-glucopyranosyluronic acid)-D-galactose
		Oligosaccharide	6-O-(4-O-methyl-D-glucopyranosyluronic acid)-D-galactose.
*L. edulis* [52]	Root bark	Phenolic lipid	Cardonol 7
		Phenolic lipid	Cardonol 13
		Cyclohexenone	5-[14-heptadecenyl]-4,5-dihydroxy-2-cyclohexenone
		Cyclohexenone	5-[16-nonadecenyl]-4S,5S-dihydroxy-2-cyclohexenone
		Cyclohexenone	5-[16-Nonadecenyl]-4,5-dihydroxy-2-cyclohexenone.
*L. humilis* [53]	Bark	Dicarboxylic acid	Malic acid
		Hydroxycinnamic acid	Quinic acid
		Gallotannin	Gallic acid glucoside
		Flavan-3-ol	(Epi)gallocatechin
		Flavan-3-ol sulfate ester	(epi)gallocatechin 5-O-methyl 7-O-sulphate
		Flavan-3-ol	(Epi)catechin
		Flavan-3-ol gallate	(Epi)-gallocatechin gallate
		Flavan-3-ol sulfate ester	3-flavan 3-,4-,5- trihydroxy5-O-methyl 7-O-sulphate
		Sulfated phenolic acid	Syringic acid sulphate
		Flavan-3-ol sulfate ester	(epi)catechin 5-O-ethyl 7-O-sulphate-3-O-hexoside
		Flavan-3-ol sulfate ester	(epi)catechin 5-O-ethyl 7-O-sulphate
		Flavan-3-ol gallate	Procyanidin dimer mono gallate
		Flavan-3-ol gallate sulfate ester	(epi)gallocatechin gallate 5-O-ethyl 7-O- sulphate.
*L. rivae* [46,54]	Root	Carotenoid	E-lutein
		Flavan-3-ol gallate	(-)-epicatechin-3-O-gallate
		Flavonol	Myricetin
		Phenol derivative	3-nonadec-14′-Z-enyl phenol
		Phenol derivative	3-heptadec-12′-Z-enyl phenol
		Phenol derivative	3-pentadec-10′-Z-enyl phenol
		Phenol derivative	3-pentadecyl phenol
		Furanone	4,5-dihydroxy-4,5-furan-2′-[16′-(Z)-18′-(E)-heneicosenyldiene] cyclohex-2-enone
		Cyclohexanone	2,4,5-trihydroxy-2-[16′-(Z)-heneicosenyl] cyclohexanone
		Cyclohexenone	4S,6R-dihydroxy-6-(12′(Z)-heptadecenyl) 2-cyclohexenone
		Cyclohexenone	4S,6R-dihydroxy-6-(14′(Z)-nonadecenyl) 2-cyclohexenone
		Cyclohexane	1,2,4-trihydroxy-4-[16′(Z)-heneicosenyl] cyclohexane.
		Sterol glycoside	Sitosterol glucoside
		Triterpenoid	Β-sitosterol
		Triterpenoid	Taraxerol
		Triterpenoid	Taraxerone
*L. schimperi* [54,55,56]	Whole plant	Phenol derivative	3-[12′(E)-pentadecenyl] fenol
		Phenol derivative	3-[14′(E)-heptadecenyl] fenol
		Phenol derivative	3-[16′(E)-nonadecenyl] fenol
		Phenol derivative	3-[18′(E)-heneicosenyl] fenol
		Cyclohexenone	5-[12′(E)-pentadecenyl] 4,5-dihydroxyciclohex-2-enone
		Cyclohexenone	5-[14′(E)-heptadecenyl] 4,5-dihydroxyciclohex-2-enone
		Cyclohexenone	5-[16′(E)-nonadecenil] 4,5-dihydroxyciclohex-2-enone
		Cyclohexenone	5-[18′(E)-heneicosenyl] 4,5-dihydroxycyclohex-2-enone
		Cyclohexenol	1-[12′(E)-pentadecenyl] cyclohex-3-en-1,2,5-triol
		Cyclohexenol	1-[14′(E)-heptadecenyl] cyclohex-3-en-1,2,5-triol
		Cyclohexenol	1-[16′(E)-nonadecenyl] cyclohex-3-en-1,2,5-triol
		Cyclohexenol	1-[14′(E)-heptadecenyl] 4-cyclohex-4-en-1,3-diol
		Cyclohexenol	1-[16′(E)-nonadecenyl] 4-cyclohex-4-en-1,3-diol
		Cyclohexenol	1-[18′(E)-heneicosenyl] 4-cyclohex-4-en-1,3-diol.
	Leaf	Lipid	Ceramide
		Alkaloid	Forsskamide
		Isoprenoid	A-tocopherol
		Triterpenoid	Betulinic acid
		Triterpenoid	Lupeol
		Triterpenoid	Oleanolic acid
		Triterpenoid	23-hydroxyoleanolic acid.
*L. schweinfurthii* [46]	Root	Phenol derivative	3-[tridecyl] phenol
		Phenol derivative	3-[heptadecyl] phenol
		Phenol derivative	3-[heptadec-12′(Z),14′(E)-dienyl] phenol
		Phenol derivative	3-[nonadec-14′(Z),16′(E)-dienyl] phenol
		Phenol derivative	3-[heneicos-16′(Z),18′(E)-dienyl] phenol
		Flavan-3-ol	Catechin
		Flavan-3-ol	Epicatechin
		Favonol rutinoside	Rutin
		Triterpenoid	Lupenone
		Cyclohexenol	1-[tridecyl] cyclohex-3-en-1,2,5-triol
		Cyclohexenol	1-[heptadecyl] cyclohex-3-en-1,2,5-triol
		Cyclohexenol	1-[tridecyl] cyclohex-4-en-1,3-diol
		Cyclohexenol	1-[nonadecyl] cyclohex-4-en-1,3-diol
		Cyclohexenol	1-[heneicosyl] cyclohex-4-en-1,3-diol
		Cyclohexenol	1-[tricosyl] cyclohex-4-en-1,3-diol
		Cyclohexenol	1-[pentadec-12′(E)-enyl] cyclohex-4-en-1,3-diol
		Cyclohexenol	1-[nonadec-14′(Z),16′(E)-dienyl] cyclohex-4-en-1,3-diol
		Cyclohexenol	1-[heneicosen-16′(Z),18′(E)-dienyl] cyclohex-4-en-1,3 diol
		Cyclohexenone	5-hydroxy-5-[tridecyl] cyclohex-2-enone
		Cyclohexenone	5-hydroxy-5-[pentadecyl] cyclohex-2-enone
		Cyclohexenone	5-hydroxy-5-[heptadecyl] cyclohex-2-enone
		Cyclohexenone	5-hydroxy-5-[pentadec-12′(E)-enyl] cyclohex-2-enone
*L. velutina* [57,58,59]	Root bark	Flavan-3-ol	Catechin (as starting unit)
		Flavan-3-ol	Epicatechin (as an extender unit).
	Leaf	Phenolic lipid	Anacardic acid
		Phenolic acid	Gallic acid
	Flower	Sesquiterpenoid	Beta-caryophyllene 22 to 36%
		Alkane	Heneicosane 4 to 10%.
*L. welwitschii* [42,60]	Whole plant	Phenolic compound	Lanneaquinol
		Phenolic compound	2′(R)-hydroxylanneaquinol.
	Leaf	Flavonol	Mearnsetin
		Flavonol glycoside	Myricetin 3-O-β-D-arabinofuranoside
		Flavonol glycoside	Myricetin-3-O-β-D-glucuronic acid
		Flavonol glycoside	Myricetin-3-O-β-D-xylofuranoside
		Flavonol glycoside	Myricetin-3-O-β-D-galactopyranoside

### 2.4. Biological Studies

Biological studies were conducted in vitro and in vivo using extracts prepared with different plant parts of *Lannea* species using, namely, the aerial part, bark, leaf, stem, root, stem and root bark, and the whole plant (Table 5). Most plant extracts were prepared with methanol or ethanol as solvents, and the bark and leaf of *Lannea* species were the most frequently used plant parts.

*L. acida* stem bark aqueous extract showed anti-diarrhoeal and anti-inflammatory activity-inhibition of prostaglandin E2 in the paw oedema method [61]; hydroalcoholic extract of the bark and the whole plant showed in vitro antioxidant activity and cytotoxic and anti-*Mycobacterium tuberculosis* H37Rv activities [62,63]; ethanolic extract of *L. acida* bark revealed in vitro antibacterial properties against Gram-negative (*Escherichia coli* and *Pseudomonas aeruginosa*) and Gram-positive (*Staphylococcus aureus*, *Enterococcus faecalis*, *Streptococcus pyogenes*, and *Bacillus subtilis*), including against resistant antibiotic strains and also oestrogenic activity and anti-osteoporotic potential in the ovariectomized Wistar rat model [64].

The in vitro antibacterial activity against *S. aureus* and antioxidant activity exhibited by *L. alata* were attributed to the presence of prenylated flavonoids, epicatechin gallate, betamidine, and myricetin [47].

Quantitative evaluation of the inhibitory (MIC) and bactericidal (MBC) concentrations of methanolic extracts of the bark, stem, and root of *L. barteri*, against *S. aureus*, *Staphylococcus epidermidis*, *Proteus mirabilis*, *E. faecalis*, *E. coli*, and *P. aeruginosa*, confirmed that this medicinal plant has significant antibacterial and antifungal activities [65,66,67].

The biological properties of *L. coromandelica* are numerous (Table 5). A stem bark extract has shown in vitro antimicrobial, hypotensive, and sporicidal activities [68]; studies on the bark revealed in vivo anti-diarrhoeal activity and in vitro antimicrobial activities [69,70,71]; the presence in stem bark of dihydroflavonols and terpenoids, polyphenols, flavonoids, kaempferol, and quercetin provided in vivo hepatoprotective and antioxidant activities to this medicinal plant [72].

According to Sohni et al., 1995, *L. edulis* whole plant water extract showed low in vitro mutagenic activity against *Salmonella typhimurium* and antioxidant activity [73].

Ethanolic and methanolic extracts of different parts of *L. velutina* showed selective in vitro antimicrobial activity against *Cladosporium cucumerinum* and *Candida albicans*; larvicidal against *Aedes aegypti*, *Anophelis gambiae*, and *Culex quinquefasciatus*; molluscidal against *Biomphalaria glabrata*, *Biomphalaria pfeifferi*, and *Bulinus truncatus;* and antioxidant activity; lipophilic root bark and hydroalcoholic stem extracts showed in vivo antioxidant and 15-lipoxygenase inhibitory activities [57,58,74].

In vitro decoction of *L. nigritana* leaf showed selective antimicrobial activity against seven reference strains and clinical isolates of *M. ulcerans* [63].

Root and stem extracts of *L. rivae* containing 2,4,5-trihydroxy-2-[16′-(*Z*)-heneicosenyl] cyclohexanone and 4,5-dihydroxy-4,5-furan-2’-[16′-(*Z*)-18-(*E*)-heneicosenyldiene] cyclohex-2-enone as marker compounds showed significant in vitro cytotoxicity in human tumour cell lines; root and stem hexane and dichloromethane extracts showed antibacterial activity against *E. faecalis* and *S. aureus*; dichloromethane/methanol (1:1) root extracts and the isolated compounds epicatechin gallate and (4*R*, 6*S*)-4,6-dihydroxy-6-((*Z*)-nonadec-14′-en-1-yl)cyclohex-2-en-1-one reduced carrageenan-induced oedema [36,54,73,75].

According to Mikail H. et al., 2016, *L. schimperi* methanolic leaf extracts demonstrated in vitro and in vitro anticoccidial activities [37,76].

Methanol, hexane, and ethyl acetate stem bark extracts of *L. schweinfurthii* showed significant in vitro antimicrobial activity against *C. albicans*, *B. subtilis*, *E. coli*, *P. aeruginosa*, and *S. aureus* [77]. In vitro acetylcholinesterase inhibitory activity (ACHE) was exhibited by a root ethyl acetate extract (IC_50_ = 0.3 ± 0.0 µg/mL), as being higher than that of galantamine control (0.53 µg/mL) [78].

*L. velutina* bark and leaf ethanolic extracts showed antioxidant and antimicrobial in vitro activities. Anacardic acid has previously been identified as one of the major compounds present in this medicinal plant [57,58,62,74,79,80].

*L. welwitschii* was also the object of different biological activity studies, like analgesic, in which the total analgesic effect of the hydroethanolic stem bark extract significantly increased in a dose-dependent manner; antibacterial activity was observed for the methanolic leaf extract against *Enterococcus faecalis*, *Klebsiella pneumoniae*, *Proteus mirabilis*, *P. aeruginosa*, *Staphylococcus aureus*, *and Escherichia coli* strains resistant to pefloxacin, with MIC values of 5, 10, 5, 2.5, and 2.5 mg mL^−1^, respectively against *E. coli*, *P. aeruginosa*, *S. aureus*, and *B. subtilis* compared to ciprofloxacin (0.025, 0.055, 0.025, and 0.02 mg/mL); antioxidant activity was exhibited by the free radical scavenging method (2,2-diphenyl-1-picrylhydrazyl (DPPH)), with an IC_50_ value of 81.8 µg/mL compared to that of α-tocopherol (1.5 µg/mL); antidiarrhoeal activity was observed for the bark aqueous extract (50–400 mg/kg), with a significant (*p* < 0.05) delay in the onset of profuse diarrhoea and reduction in intestinal fluid volume; anti-inflammatory activity at 200 mg/kg dose had an inhibition of 14.49 ± 2.43% compared to the control in the paw oedema method, while the total oedema induced over the 6 h was 37.19 ± 4.38%. The maximum inhibitory effects were verified with a dose of 400 mg/kg. Myricetin, a common phenolic compound present in several plants, has previously been identified in *L. welwitschii* [60,81,82].

*L. acida* was the most studied *Lannea* species, followed by *L. coromandelica* and *L. velutina*. Different biological activities were observed, but the predominant ones were by far antimicrobial, antioxidant, and anti-inflammatory activities.

**Table 5:** In vitro and in vivo biological studies on *Lannea* species.

Species	Plant Part	Extract	Test	Results	Refs
*Lannea acida*	Wp	EtOH	In vitro: antibacterial activity	Potential source of new antibacterial agents against Gram-negative (*Escherichia coli* and *Pseudomonas aeruginosas*) and Gram-positive (*Staphylococcus aureus*, *Enterococcus faecalis*, *Streptococcus pyogenes* and *Bacillus subtilis*); crude extract showed bactericidal and bacteriostatic activity (IC_50_ values between 12 and 94 µg/mL).	[64]
	Wp	H_2_O, MeOH	In vivo: reproductive toxicity of colibri in adult male rats	Treatment with *L. acida* extracts was significant (*p* ≤ 0.05–0.001) because it reversed the reproductive system-induced damage, especially after 28 days of treatment with aqueous solution (340 mg/kg) and methanol extracts (170 mg/kg).	[83]
	Wp	EtOH	In vivo: antibacterial activity by microdilution in broths of bacterial strains	Selective antibacterial activity against Gram-negative (*E. coli* and *P. aeruginosa*) and Gram-positive (*S. aureus*, *E. faecalis*, *S. pyogenes*, and *B. subtilis*), including against resistant strains, with MICs/MBCs ranging from 7.80 to 125 µg/mL. The highest sensitivity was seen against *Bacillus subtilis* and *Pseudomonas aeruginosa*.	[62]
	B	EtOH	In vitro: Folin Method–Ciocalteu (antioxidant activity)	Determination of total phenolic compounds and flavonoids by the Folin Ciocalteu method, expressed in mg of gallic acid equivalents and quercetin equivalents, respectively (total phenols vary between 34.4 to 40.55; total flavonoids vary between 6.4 and 11.02).	[40]
	B	EtOH	In vitro and in vivo: evaluation of oestrogenic activity and anti-osteoporotic potential in ovariectomized Wistar rats	*L. acida* bark extract induced proliferation of MCF-7 cells. At 200 mg/kg, prolonged treatment with the extract prevented ovariectomy-induced body weight gain and loss of bone mass and/or density. The ethanol extract induced a significant increase in MCF-7 cell production at concentrations of 10 (*p* < 0.05), 100 (*p* < 0.05), and 200 (*p* < 0.01)/g/mL compared to control DMSO.	[84]
	StB	Hx, Chl, Ace	In vitro: antimicrobial activity	The antimicrobial test result showed that stem bark extracts exhibited antimicrobial activity against several microorganisms (*Bacillus cereus*, *Escherichia coli*, *Klebsiella pneumonia*, *Pseudomonas aeruginosa*, *Staphylococcus aureus*, and *Streptococcus pyogenes*), with clear zones of inhibition ranging from 6 mm to 21 mm.	[85]
	StB	H_2_O	In vivo: anti-inflammatory activities by method PGE E-2-induced paw oedema	The extract inhibited paw oedema significantly (F(3,96) = 25.02; *p* < 0.05) and (F(5,96) = 16.46; *p* < 0.01) at doses of 100 mg/kg and 300 mg/kg, respectively. However, the extract did not show significant inhibition at 30 mg/kg (F(15,96) = 1.12; *p* = 0.3505). Aqueous extract inhibited prostaglandin E2-anti-inflammatory activity.	[61]
	B	EtOH	In vitro: antioxidant activity by DPPH	Antioxidant activity through DPPH method using quercetin and gallic acid as positive controls. The IC_50_ value of each extract was determined and all tests were performed in triplicate. The bark extract of *Lannea acida* showed IC_50_ = 345.72 ± 7.76 μg mL^−1^ while that of *Lannea velutina* IC_50_ = 478. 68 ± 8.55.	[40]
	R B	DCM	In vitro: antiproliferative activity	The XTT assay was used to evaluate the antiproliferative activity of the extract, fractions, and compounds on three multiple myeloma cell lines: RPMI 8226, MM.1S, and MM.1R. Fractions were considered active when they inhibited at least 50% of cell growth at 20 μg/mL; two compounds showed activity on all cell lines with IC_50_ values < 5 µM. Bortezomib was used as a positive control.	[44]
	Wp	EtOH	In vitro: cytotoxic and anti-Mycobacterium tuberculosis H37Rv activities	The rate of monocytes at different stages of mitosis was corrected in the absence and presence of the extract as follows: G0/G1 58.83–59.83%; synthesis 21.95–18.64%; mitosis 16.67–15.97%; necrosis 2.65–5.64%. The percentage of inhibition of *Mycobacterium tuberculosis* proliferation was 77.6 and 36.8%, respectively, for 1.2 and 0.6 mg mL^−1^ of extract.	[62]
*Lannea barteri*	L and St	MeOH	In vitro: antibacterial activity using the agar well diffusion method	MBC determination showed that the MBC ranges for methanolic and ethanolic extracts of *L. barteri* leaves were 6.25 to 50 mg/mL and 6.25 to 12.5 mg/mL, respectively. The rapid death of *S. aureus* was verified in the range of 1.45 × 10^6^ CFU of minimum bactericidal concentration (MBC) of methanolic leaf extract of *L. barteri*.	[66]
	L, StB	DCM, MeOH, H_2_O	In vitro: anticancer activity	The extracts and fractions were tested for anticancer activity by using the crystal violet cell proliferation on four adherent human carcinoma cell lines. The inhibitory concentration (IC_50_) of fractions IH, 1I, 2E, and 2F were: 3.75 ± 1.33, 3.88 ± 2.15, 0.53 ± 0.41, and 0.42 ± 0.45 µg/mL against KYSE 70 and 1.04 ± 0.94, 2.69 ± 1.17, 2.38 ± 3.64, and 2.17 ± 1.92 µg/mL against SiSo cell lines, respectively. Fraction 2E showed weak apoptotic activity at double the IC_50_ and some sign of cell cycle arrest in the G2/M phase	[86]
*Lannea coromandelica*	L	EtOAc, MeOH, H_2_O	In vitro: antioxidant activity by DPPH method	The ethyl acetate fraction had stronger DPPH scavenging activity than the methanolic extract and aqueous extract fractions. The DPPH clearing effect of both standards and plant extracts occurred in the order of BHT > EAF > CME > AqF and was 91.9%, 71.4%, 56.2%, and 42.2% at a concentration of 100 µg/mL, respectively.	[87]
	Wp	EtOH	In vivo: hypotensive activity	The ethanolic extract of *L. coromandelica* was administered to dogs and rats at doses 5–100 mg/kg and 1–25 mg/kg, respectively, and a reduction in blood pressure was observed.	[88]
	L	EtOH:H_2_O	In vivo: anti-ulcer activity model	*L. coromandelica* anti-ulcer activity was evaluated in two different in vivo models of induced gastric ulcer. Leaf hydroethanolic extract showed significant levels of ulcer inhibition and gastric protection.	[89]
	L	MeOH	In vitro: neuropharmacological and antidiabetic activity	Rats received doses of 100, 150, and 200 mg/kg of body weight in an elevated plus maze and motor coordination; 100 and 200 mg/kg of body weight in sleep time, hole crossing, hole plate, and open field testing; and 200 and 400 mg/kg body weight in the antidiabetic activity test. The results obtained were all significant and dose dependent. *L. coromandelica* extracts possess significant neuromodulatory properties, had no significant effect on normal blood sugar levels, but corrected alloxan-induced changes in blood sugar and pancreas.	[90]
	B	MeOH	In vitro: antioxidant activity by DPPH method	The percentage of free radical scavenging by the DPPH, with IC_50_ 12.12 ± 0.48 µg/mL compared to the ascorbic acid standard 8.66 ± 0.11 µg.	[84]
	L	EtOH	In vitro: antidiabetic activity in rats	Blood glucose levels in normal rats reached high levels 60 min after oral glucose administration (3 g/kg) and gradually decreased to 125 mg/dL in 2 h. Groups pretreated with ethanolic extract of *L. coromandelica* (100 and 200 mg/kg) and metformin (250 mg/kg) had induced decreased blood glucose levels significantly (*p* < 0.05) compared with that of the control group.	[56]
	B	MeOH	In vivo: castor oil-induced antidiarrhoeal activity	The extract considerably reduced the number of diarrhoeal episodes compared to control animals. The bark extract of *L. coromandelica* at a dose of 200 mg/kg showed a significant reduction (*p* < 0.05) of 68.86% in the number of faecal episodes, compared to the antidiarrheal drug, loperamide which has 89, 14% protection.	[69]
	L	MeOH	In vivo: aspirin-induced antiulcer activity	The test was performed on albino rats weighing between 150 and 200 g, using an aqueous suspension of aspirin at a dose of 200 mg/kg orally for 8 days. The result was a significant decrease in the ulcer index, with the percentage of gastric protection of 17.3% (standard), 78.29% (positive control), 30.57% (low dose), and 62.76% (high dose), and a significant reduction in the volume of gastric juice and acidity and increase in pH.	[91]
	B	MeOH	In vitro: antibacterial activity	Methanolic extract of *L. coromandelica* revealed a significant moderate antibacterial activity against *Staphylococcus aureus*, *Salmonella typhi*, *Shigella dysenteriae*, *Pseudomonas aeruginosa*, and *Escherichia coli*; there was no activity against *Shigella boydii*, however, there was a greater zone of inhibition against *Escherichia coli* (inhibition zone of 15.59 ± 0.22 mm), followed by Staphylococcus aureus and *Shigella dysenteriae*.	[69]
	B	EtOH	In vivo: thioacetamide-induced hepatoprotective and antioxidant activity in rats	Hepatotoxicity was induced by thiocetamide 100 mg/kg subcutaneously in male Wistar rats, causing marked changes in serum AST, ALT, ALP, and serum bilirubin and reduced serum concentration of total proteins, albumin, sodium, and potassium compared to those in the control (*p* < 0.05). The results showed that the hydroalcoholic extracts of the bark of *L. coromandelica* used at a higher dose (400 mg/kg) reduced AST ((138 ± 5.1) IU/L) to the maximum ((71 ± 5.1) IU/L), ALT ((71 ± 2.7) IU/L), ALP ((140 ± 1.9) IU/L), and serum levels of bilirubin, cholesterol, sugar, and LDH.	[72]
	L	EtOH	In vivo: antidiabetic activity in rats induced by alloxan	The ethanolic extract of *L. coromandelica* (100 to 200 mg/kg) reduced the glucose level (123 ± 2.2 and 115 ± 2.6, respectively) both in diabetic animals and in those induced with alloxan when compared to normal animals (74 ± 1.7 and 70 ± 1.4).	[92]
	R	EtOH	In vitro: antioxidant activity	The crude extract of ethyl acetate at concentrations 200; 100; 50; 25; 12.5; and 6.25 µg/mL, in 3 mL of methanolic DPPH solution. Ascorbic acid was used as a positive control. The compound isolated from the extract (citrinin) showed moderate antioxidant activity (AAI 0.671 and IC_50_ 145.9 ppm).	[93]
	Wp	EtOAc	Antimicrobial activity agar diffusion method	The antimicrobial activity demonstrated that the isolated compound was not active against *Escherichia coli* ATCC25922, *Salmonella typhi* ATCC 14028, *Staphylococcus aureus* ATCC25923, and *Pseudomonas aeruginosa* ATCC 27853 (MIC: 1000 μg/mL).	[93]
*Lannea edulis*	Wp	H_2_O	In vitro: mutagenicity test	The mutagenicity test was performed using *Salmonella typhimurium* strains TA97a, TA98, and TA100, and marginal-type displacement mutations (marginal mutagenicity) were observed in the TA97a strain.	[73]
	L	H_2_O	Antidiabetic activity by alloxan induction method	Daily dosing of *L. edulis* resulted in significant reductions in blood glucose levels compared to those in the diabetic control from day 3; only the 300 mg/kg and 500 mg/kg *L. edulis* diabetic positive control groups had significant differences (*p* < 0.05) in mean blood glucose levels. The 100 mg/kg diabetic positive control group kg of *L. edulis* showed significant difference (*p* < 0.05) compared to diabetic control group from day 5.	[75]
		H_2_O	In vitro: cytotoxic activity	The cytotoxic effect of aqueous extracts was evaluated on U937, MeWo, and Vero cell lines tested. *L. edulis* at the highest tested concentration was seen to be significantly toxic (*p* = 0.007). *L. edulis* (*p* < 0.007) showed a similar toxic effect in the MeWo and Vero cell lines.	[94]
	Wp	H_2_O	In vitro: anti-inflammatory activity	The anti-inflammatory potential of the extract was evaluated on RAW 264.7 cells, and there was no anti-inflammatory activity observed for the plants tested. However, in the absence of LPS stimulation, there was an increase of NO production, indicating that the extracts might have pro-inflammatory properties.	[94]
*Lannea humilis*	B	MeOH	In vitro: antioxidant activity by DPPH and FRAP methods	DPPH = 9.3 (EC50 µg/mL); FRAP = 19.77 (mM FeSO_4_ equivalent/mg sample).	[53]
	Stem bark	MeOH	In vitro: antioxidant activity by DPPH method	The antioxidant activity of plant extracts demonstrated dose-dependent behaviour. The ethyl acetate extract displayed the most noteworthy antioxidant activity of 98% at 240 µg/mL, followed by the hexane extract with antioxidant activity of 92% at 240 µg/mL. Methanol extract showed antioxidant activity of 71% at 240 µg/mL.	[95]
*Lannea nigritana*	R	H_2_O	In vitro: proportional method for MIC determination	Leaf decoction showed activity on 7 *M. ulcerans* strains and isolates with mean MIC values of 40 μg/mL.	[63]
	StB	EtOH	In vitro: cytotoxic activity of the ethanolic extract by the HeLa method	Extracts can be classified as being of low cytotoxicity, showing less than 40% activity at 500 µg/mL.	[96]
*Lannea rivae*	B	DCM/MeOH	In vivo: anti-inflammatory activity by method paw oedema in Wistar rats	Extract of *L. rivae* roots and epicatechin gallate and (4R, 6S)-4,6-dihydroxy-6-((Z)-nonadec14’-en-1-yl)cyclohex-2-en-1 -one at 200 mg/kg using Indomethacin as the standard showed anti-inflammatory activity; both the extract and the 2 compounds moderately inhibited the oedema induced by carrageenan, however, none of them reached the level of inhibition of the Indomethacin standard.	[36]
	R	DCM/MeOH	In vitro: antibacterial activity	The new compounds isolated (4R,6S)-4,6-dihydroxy-6-((Z)-nonadec-14′-en-1-yl)cyclohex-2-en-1-one and (2S*,4R*,5S*)-2,4,5-trihydroxy-2-((Z)-nonadec-14′-en-1-yl)cyclohexanone were tested against *Staphylococcus aureus* and *Escherichia coli*. Compound 1, taraxerol, β-sitosterol, taraxerone, and lupeol showed moderate activity against *E. coli* (56.64% inhibition), while only compound 2 and β-sitosterol showed activity against *S. aureus* (43.56%).	[36]
	R, St	Hx, DCM, EtOAc, MeOH	In vitro: antibacterial activity of selected compounds	The hexane extracts of *L. rivae* exhibited intermediate antibacterial activity against *E. faecalis*, while the DCM extracts showed intermediate activity against both Gram-positive bacteria *E. faecalis* and *S. aureus*, but no activity against Gram-negative bacteria. The EtOAc and MeOH extracts demonstrated a broader spectrum of activity, with better activity being observed with the Gram-positive bacteria.	[46]
*Lannea schimperi*	Ap	EtOH	In vivo: effect of ethanolic extract on ethanol/HCl-induced gastric ulcers in rats	Doses of ethanolic extract of 100, 200, 400, and 800 mg/kg were tested in rats against gastric ulcer induced by ethanol-HCl and the effects were compared to those of pantoprazole 40 mg; after removal and analysis of the stomach, it was found that the ethanolic extract of *L. schimperi* showed an average protection of 81.7% compared to 87.5% for the drug pantoprazole.	[55]
	L	MeOH	In vitro: anticoccidial activity in Eimeria tenella oocysts	This activity was carried out using oocysts isolated from infected chicks, and three doses of methanolic extract of *L. schimperi* leaves were used, 25 mg/mL, 50 mg/mL, and 100 mg/mL. Anticoccidial activity was determined by counting lysed and non-sporulated oocysts and sporulated oocysts. The extract dose at 100 mg/mL exhibited 98% higher anticoccidial activity and an inhibition of 97.92%. Doses 25 and 50 mg/mL of extract showed activities and inhibitions against non-sporulated oocysts of *E. tenella* of 68% and 89% and 66.65 and 88.5, respectively.	[37]
	R, St	MeOH, H_2_O	In vitro: cytotoxic activity colorimetric test	MTT was used to measure all growth and cellular chemosensitivity. The samples were prepared for a stock solution of 20 mg/mL in 100% DMSO, and emetine was used as a positive control. The 5-[alkenyl]-4,5-dihydroxycyclohex-2-enone mixture (1a-d) exhibited good in vitro cytotoxicity against the Chinese Hamster Ovarian mammalian cell line.	[97]
	MeOH	MeOH	In vivo: anti-inflammatory activity	The test was carried out using the egg albumin induction method in rats. Tested doses were 12 and 24 mg/kg, and acetylsalicylic acid 80 mg was used as standard. The anti-inflammatory response was significant (*p*< 0.05); however, there was no significant difference (*p* > 0.05) between the extract-treated groups and the standard drug-treated group (positive control).	[98]
*Lannea schweinfurthii*	Wp	Hx, MeOH, EtOAc	In vitro: antibacterial and antifungal activity	The extracts were tested against *S. aureus*, *Bacillus subtilis*, *P. aeruginosa*, *Escherichia coli*, and *Candida albicans*. Measured inhibition zone showed significant differences: 7 mm hexane extract (α = 0.05); methanolic and ethyl acetate showed high activity (13 mm inhibition and above). Both extracts showed moderate activity, with inhibition between 7 and 14 mm against bacteria and fungi.	[77]
	R	EtOAc	In vitro: ACHE inhibitory activity	The ethyl acetate extract of *L. schweinfurthii* showed an IC_50_ value higher than that of galanthamine (standard) 0.00053 mg/mL. The extract has ACHE inhibitory activity with an IC_50_ of 0.0030 ± 0.000 mg/mL.	[78]
	R	Hx	In vitro: antibacterial activity	The extract was active against *Enterococcus faecalis* and *Enterococcus faecium* with10 mm zone of inhibition.	[31]
	R, St	MeOH	In vitro: antibacterial activity	Active against *Salmonella typhimurium*, *Enterococcus faecalis*, *Enterococcus faecium*, *Pseudomonas aeruginosa*, and *Staphylococcus aureus* with zone of inhibition ranging from 8 mm to 15 mm.	[31]
	B	MeOH	In vitro: anti-HIV-2 activity	The methanolic extract of the stem bark of *L. Schweinfurthii* was active against HIV type 2, with IC50 values < 10 µg/mL and 9.9 µg/mL against HIV-1, respectively.	[99]
*Lannea velutina*	R B	MeOH, EtOH	In vitro: DPPH radical scavenging activities and 15-LOX inhibition	The concentrations of extracts and fractions that provide 50% radical scavenging are (12 ± 2 and 17 ± 2) and 50% enzyme inhibition (14 ± 1 and 18 ± 2), respectively; scavenging activity and inhibitory effect were statistically very significant; *p* < 0.001.	[74]
	R B	EtOH:H_2_O	In vitro: antioxidant activity DPPH method	50% radical scavenging, at concentrations of 5–7 micrograms/mL, and 15-lipoxygenase inhibitors (50% inhibition at 10–18 micrograms/mL). *L. velutina* extract possessed a weak DPPH radical scavenging action.	[40]
	Wp	EtOH, DCM, MeOH, H_2_O	In vitro. Antimicrobial activity tested on mosquito larvae; molluscicidal activity with molluscs	Positive results were obtained for antioxidant activity (methanolic extracts of bark and roots), antifungal activity (dichloromethane extract active against *Candida albicans* and *Cladosporium cucumerinum)*; larvicidal activity against the malarial mosquito *Anopheles gambiae* (dichloromethane extract of bark and methanolic extract of leaves); and molluscicidal activity directed at the snail *Biomphalaria pfeifferi*, transmitter of schistosiasis. The ethanol extract of the bark showed greater antibacterial activity against *Bacillus subtilis*, *Staphylococcus aureus* (Gram-positive), *Pseudomonas aeruginosa*, and *Salmonella typhimurium* (Gram-negative).	[57,100]
	R B, StB	EtOH, MeOH, H_2_O	In vitro: antioxidant activity by DPPH method	Petroleum ether, chloroform, and dichloromethane extracts are inactive as DPPH radical scavengers; the aqueous extract had moderate activity while the methanolic and hydroalcoholic extracts of root bark and stem bark were very active.	[57]
	B	EtOH	In vitro: antioxidant activity by DPPH method	For the test on the free radical potential on the radical DPPH, o *L. velutina*, which showed a percentage inhibition of 52.8125 ± 2.16% lower than that of the gallic acid, was used as reference substance.	[79]
	B	EtOH	In vitro: antimicrobial activity by inhibition method	*Shigella dysenteria*, *S. aureus* were sensitive to *Lannea velutina* extracts with inhibition diameters of 10 mm; Bacillus cereus and *Escherichia coli* were also sensitive to the extract with 8 mm and *Salmonella thyphi* with 7 millimetres.	[79]
	L	Hx, EtOAc, DCM, MeOH, H_2_O	In vitro: antioxidant activity by DPPH method	The *L. velutina* leaf methanol extract showed IC_50_ 15.42 g/mL.	[80]
	L	Hx, EtOAc, DCM, MeOH, H_2_O	In vivo: acute toxicity	The acute oral toxicity test of ethyl acetate, methanol, and aqueous extracts on mice exhibit a lethal dose (LD_50_) estimated to be higher than 2000 mg/kg body weight.	[80]
*Lannea welwitschii*	B	H_2_O	In vivo: anti-diarrhoeal activity in mice	Bark aqueous extract (50–400 mg/kg) caused a significant delay (*p* < 0.05) in the onset of profuse diarrhoea, decreased purging frequency, wet stool weight, and diarrhoea severity. Oral administration of castor oil produced an intestinal fluid volume of 2.33 ± 0.17 mL; Lw bark aqueous extract at 400 mg/kg significantly (*p* < 0.05) reduced intestinal fluid volume to 1.40 ± 0.25.	[60]
	B	H_2_O	In vivo: anti-diarrhoeal activity in mice	The acute toxicity tests carried out showed a well-tolerated effect of the drug via oral route, a dose of 20 g/kg produced no death in the animals. LD50 was estimated to be 631 mg/kg.	[82]
	L	MeOH	In vivo: analgesic activity	In doses of 50, 200, and 400 mg/kg, *L. welwitschii* extract caused a significant increase (*p* < 0.0001) in the mean reaction time of treated mice (49.67 ± 2.18%, 63.20 ± 2.54%, and 59.42 ± 0.84%) respectively compared to the control group, while the total analgesic effect (AUC) was significant (*p* < 0.0001) and the dose-dependent increase was to 159.20 ± 19.65, 202.30 ± 12.44 and 228.8 ± 11.29, respectively. There was no statistical difference in the analgesia produced with 100 mg/kg aspirin.	[60]
	L	MeOH	In vitro: antioxidant activity by DPPH method	MeOH extract showed antioxidant activity with IC_50_ 81.8 µg mL^−1^ compared to α-tocopherol 1.5 µg/mL.	[81]
	L	MeOH	In vitro: antimicrobial activity by agar diffusion and microdilution methods	The extract showed activity against *Enterococcus faecalis*, *Klebsiella pneumoniae*, *Proteus mirabilis*, *Pseudomonas aeruginosa*, *Staphylococcus aureus*, and some strains of *Escherichia coli* resistant to pefloxacin. The methanolic extract of *L. welwitschii* showed MICs of 5, 10, 5, 2.5, and 2.5 mg/mL, respectively, against *E. coli*, *P. aeruginosa*, *S. aureus*, and *B. subtilis* compared to Ciprofloxacin which was 0.025; 0.055; 0.025; 0.02 mg/mL while the MICs of methanolic leaf extract and clotrimazole against *C. albicans* were 2.5 and 0.025 mg/mL, respectively.	[60]
	StB	EtOH:H_2_O	In vivo: anti-inflammatory activity by method carrageenan-induced paw oedema	The *L. welwitschii* extract was administered at doses of 50, 200, and 400 mg/kg. The 200 mg/kg dose had an inhibition of 14.49 ± 2.43% compared to the control, while the total oedema induced over 6 h was 37.19 ± 4.38% The maximum inhibitory effects were seen with 400 mg/kg dose.	[60]
	Wp	DCM, MeOH	In vitro: antioxidant activity by spectrophotometric methodology	The antioxidant activity of identified Compound 4 (IC_50_ 18.6 ± 4.5 µg/mL) and 2 (IC_50_ 20.0 ± 0.1 µg/mL) showed better activity than the controls, ascorbic acid (IC_50_ 23.17 ± 2.02), and quercetin (IC_50_ 31.67 ± 2.88 µg/mL)	[42]

Aerial part—Ap; Ace—acetone; AgNps—green silver nanoparticles; AP—aerial part; Ba—bark; Be—berries; ButOH—butanol; C_6_H_14_—petroleum ether; CFU—Per milliliter colony forming unit; Chl—chloroform; DCM—dichloromethane; DMSO—dimethyl sulfoxide; Et_2_O—diethyl ether; EtOAc—ethyl acetate; EtOH—ethanol; Fl—flower; Fr—fruit; H_2_O—water; Hx—hexane; IC_50_—median inhibition concentration; Iz—inhibition zone; L—leaf; MBC—minimum bactericide concentration; MeOH—methanol; MIC—minimum inhibitory concentration; NA; Na2SO4—sodium sulfate; N-Hx—N-hexane; P—pulp; R—root; Se—seed; Sf—supercritical fluid; St—stem; StB—stem bark; StO—steam distilled oil; whole plant—Wp.

## 3. Discussion

Our analysis found that 14 *Lannea* species are reportedly used in traditional medicinal systems of over 35 countries to treat a variety of disease signals and symptoms. Among these, fever, inflammation, diabetes-related symptoms, gastrointestinal disorders, and sexually transmitted diseases are the most common diseases treated with various extracts of *Lannea* species. Although not all *Lannea* species have been studied for their biological activity, those that have been showed antimicrobial, antioxidant, and anti-inflammatory properties, mainly observed in vitro. These results support the use of *Lannea* medicinal plants in traditional medicinal systems, as most of their applications are in the treatment of disease symptoms related to the biological activities observed in vitro.

In the genus *Lannea*, some characteristic *Anacardiaceae* compounds such as anacardic acid, as well as common natural products such as gallic acid and derivatives, flavonol derivatives such as quercetin and rutin, kaempferol, myricetin, and flavones like luteolin, have been identified [49,58,97,101].

Twelve different biological activities have been reported in vitro and/or in vivo for *Lannea* species, with antimicrobial, antioxidant, anti-inflammatory, and cytotoxic activities being the most common. In many cases, the observed activity was considered significant when compared to the positive controls used in the studies. Most extracts were prepared with methanol, ethanol, and water, suggesting that most extracted compounds have a relatively high polarity.

Previous research on anacardic acid showed that this natural compound can exhibit a wide variety of other biological activities. For instance, antibacterial activity was observed against bacteria species like *Bacilus subtilis*, *Helycobacter pylori*, *Propionibacterium acnes*, and *Staphylococcus aureus*. Antimicrobial activity exhibited by *L. velutina* ethanolic leaf extracts, in which this compound has previously been identified, thus may be related to anacardic acid [100,101].

In an in vivo mouse model of inflammation induced by carrageenan, prostaglandin E2, dextran, and histamine, the effects of pretreatment with anacardic acid (administered at doses of 10, 25, and 50 mg/kg intraperitoneally) were investigated. The study revealed that anacardic acid exhibited inhibitory effects on carrageenan-induced oedema, with a significant efficacy observed at a dose of 25 mg/kg, surpassing that of the positive control, indomethacin. Histological examination of tissue specimens from the anacardic acid-treated group indicated reduced neutrophil infiltration compared to the carrageenan-treated group. Furthermore, anacardic acid demonstrated inhibitory properties against carrageenan-induced depletion of glutathione and reduced levels of malondialdehyde, a pivotal marker of oxidative stress. Taken together, these results suggest that the anti-inflammatory effect of anacardic acid is due to its ability to inhibit inflammatory mediators, mitigate chemotaxis, and alleviate oxidative stress. In addition, the assessment of antinociceptive activity showed a reduction in pain symptoms in the anacardic acid-treated group. Mechanistic insights into this activity revealed a link to opioid receptors, as demonstrated using the nonselective opioid receptor antagonist naloxone as a control [102].

Anacardic acid also exhibited modulatory activity in gene expression, cell death, and cell proliferation; selective cytotoxicity against human cancer cell lines was also observed, indicating that this compound may be a useful focus of study for the development of new therapeutic anticancer agents [101].

Quercetin, a common flavonol abundantly present in numerous plant species, has a significant antioxidant activity and has been described to prevent diseases like osteoporosis, cancer, tumours, and lung and cardiovascular diseases. In vivo studies have shown that this antioxidant activity is mainly exerted through the effect on gluthathione reactive oxygen species, enzymatic activity (namely acetylcholinesterase), and signal transduction pathways. Quercetin has also shown to be able to prevent lipopolysaccharide (LPS)-induced heart damage by clearing oxygen-free radicals and consequently preventing myocardium damage. Its activity is also exerted in several steps of signal transduction pathways, decreasing the impact of oxidative stress. In a LPS-induced acute liver injury in vivo mouse model, quercetin inhibited NF-κB and MAPK signalling pathways and inhibited the expression of apoptosis-related proteins, which led to decreased oxidative stress and tissue damage. Antioxidant and anti-inflammatory properties have been demonstrated for *L. acida* and *L. coromandelica*, from which quercetin has previously been identified [103]. This natural product demonstrated selective in vitro antibacterial efficacy against various infectious strains of both Gram-positive and Gram-negative bacteria. Notably, *Staphylococcus aureus*, *Staphylococcus epidermidis*, and clinical strains of methicillin-resistant *Staphylococcus aureus* (MRSA) exhibited significant susceptibility to quercetin. Furthermore, when administered concomitantly with antibiotics such as ampicillin, erythromycin, gentamycin, oxacillin, and vancomycin, quercetin significantly potentiated the antibacterial activity of these drugs against clinical MRSA strains, implying a synergistic interaction between quercetin and antibiotics. This observed phenomenon underscores the potential of quercetin as a promising therapeutic agent for the treatment of infectious diseases [104].

In other antibacterial studies, quercetin has showed inhibitory activity on pathogenic bacteria growth, namely *E. coli*, *P. mirabilis*, *Aspergillus flavus*, *P. aeruginosa*, *Salmonella enteritidis*, and *S. aureus*. Synthetic derivatives of this compound also showed growth inhibitory activity against *E. coli*, *S. aureus*, and *P. aeruginosa*. The current research proposes that the antibacterial mechanism is related to cell wall destruction and cell permeability deregulation, compromising metabolic pathways crucial for bacterial survival, like protein synthesis and expression, enzyme activity, and nucleic acid synthesis. This mechanism may justify the synergistic effect observed when quercetin was administered in combination with antibiotics [105].

Myricetin is a flavonol with a wide distribution in many plants and is highly recognised for its nutritional value. Previously conducted studies on this compound showed that it can display different biological activities, such as antioxidant activity, being able to reduce oxidative stress through mechanisms like radical scavenging, decreasing production of pro-inflammatory agents, and disrupting inflammatory pathways. Similar activities have also been observed for *L. welwitschii* and *L. rivae*, where this compound was previously identified. Anticancer activity has also been reported, with myricetin exhibiting selective cytotoxic activity against human hepatic, pancreatic, skin, colon, and leukaemia cancer cell lines with clinical relevance. Research showed that myricetin can also interfere with different mechanisms related to tumour proliferation, namely modulating gene expression and inhibiting enzymes and other agents that directly promote cell division. Other studies showed that myricetin can act as an anti-platelet aggregation agent, supressing thromboxane formation and inhibiting specific receptor binding of platelet activating factor, and as an antihypertensive agent, reducing systolic blood pressure and vascular reactivity; immunomodulatory activity has been described in vivo and in vitro, with myricetin acting on stimulating antibody formation and regulating TNF-α, IL-2, IL-6, and IL-12 expression and lymphocyte proliferation [106].

Flavonoid compounds like catechins and its derivatives, found in *L. alata*, and terpenoid compounds like *b*-sitosterol, found in *L. coromoandelica*, have previously been studied for their biological activities. While catechins have shown antioxidant activity in in vitro essays, *b*-sitosterol has exhibited several in vitro biological activities like antimicrobial, anti-inflammatory, antioxidant, and antidiabetic activities [96,107].

Understanding the biological activities of plant extracts represents a significant challenge due to their complex composition, which includes a variety of natural products derived from the secondary metabolism of plants. It is often believed that the observed activities of plant extracts are associated with the presence of the most common occurring compounds or classes of compounds; however, this association often occurs based on an equilibrium between concentrations of compounds belonging to different classes. In particular, synergistic and other complex interactions may play a role, and numerous reports documented in the literature indicate that the biological activities of isolated major compounds can be inferior to those of all extracts.

Our research showed that different plant parts of *Lannea* species are used as medicinal plants for the preparation of traditional herbal preparations through decoction and maceration. Phytochemical studies on this genus have shown that phenolic compounds are the chemical class with higher representativity, and that *Lannea* species have in vitro/in vivo biological activities (antibacterial, antidiabetic, antifungal, antimicrobial, anti-inflammatory, antioxidant, antipyretic). Since these activities reported in the literature are aligned with their use in traditional medicine, we can thus consider that this use is totally or partially scientifically valid.

Given that a significant proportion of the identified secondary metabolites in *Lannea* species belong to the chemical class of polyphenols, it is plausible to correlate the observed biological activities with phenolic compounds in general. Nevertheless, this hypothesis requires empirical validation through specific studies aimed at a comprehensive characterization of these activities.

## 4. Materials and Methods

This review was performed following the criteria described in the Preferred Reporting Items for Systematic Reviews and Meta-Analyses (PRISMA) statement 2020 (https://prisma-statement.org/prismastatement/flowdiagram.aspx; accessed on 1 February 2023).

A literature search covering articles published between January 1995 and June 2023 was conducted using databases from, B-on, Google Schoolar, Prelude Medicinal Plants database, Pubmed, Web of science, and primary bibliographic sources. These bibliographic sources were searched using different key words: “*Lannea*”; “Ethnomedicinal”; “Chemical”; “Biological activity”, and the Boolean connectors AND/OR.

The studies that were related to plants belonging to the *Lannea* genus and were concerned with their medicinal importance were selected and included in this review.

## 5. Conclusions

*Lannea* species may represent an important source of natural products with relevant biological activities that can contribute to the development of new drugs. This study of this genus highlights its importance for traditional medicine in developing countries where access to primary health care is still poor. Despite this wide utilization, more multidisciplinary (taxonomic, conservational, ethnopharmacological) studies are needed to validate their concrete use as herbal medicines for the specific treatment of pathologies to which they are traditionally indicated.

## Figures and Tables

**Table 1:** *Lannea* A. Rich. in Guill. accepted species.

*Lannea acida* A.Rich.	*Lannea humilis* (Oliv.) Engl.
*Lannea acuminata* Engl.	*Lannea katangensis* Van der Veken
*Lannea alata* (Engl.) Engl.	*Lannea ledermannii* Engl.
*Lannea ambacensis* (Hiern) Engl.	*Lannea malifolia* (Chiov.) Sacleux
*Lannea angolensis* R. Fern. & Mendes	*Lannea microcarpa* Engl. & K.Krause
*Lannea antiscorbutica* (Hiern) Engl.	*Lannea nigritana* (Scott Elliot) Keay
*Lannea asymmetrica* R.E.Fr.	*Lannea obovata* (Hook.f. ex Oliv.) Engl.
*Lannea barteri* (Oliv.) Engl.	*Lannea rivae* (Chiov.) Sacleux
*Lannea chevalieri* Engl.	*Lannea rubra* (Hiern) Engl.
*Lannea cinerascens* Engl.	*Lannea schimperi* (Hochst. ex A.Rich.) Engl.
*Lannea coromandelica* (Houtt.) Merr.	*Lannea schweinfurthii* (Engl.) Engl.
*Lannea cotoneaster* (Chiov.) Sacleux	*Lannea tibatensis* Engl.
*Lannea discolor* (Sond.) Engl.	*Lannea transulta* (Balf.f.) Radcl. Sm.
*Lannea edulis* (Sond.) Engl.	*Lannea triphylla* (Hochst. ex A.Rich.) Engl.
*Lannea fruticosa* (Hochst. ex A.Rich.) Engl.	*Lannea velutina* A.Rich.
*Lannea fulva* (Engl.) Engl.	*Lannea virgata* R.Fern. & A.Fern.
*Lannea glabrescens* Engl.	*Lannea welwitschii* (Hiern) Engl.
*Lannea gossweileri* Exell & Mendonça	*Lannea zastrowiana* Engl. & Brehmer

Adapted from: WFO (2023)*: Lannea* A. Rich. in Guill. [7]

**Table 2:** *Lannea* species used in traditional medicine and their vernacular names, countries, and ethnic groups.

Species, (Synonyms)	Country	Vernacular Name (Ethnic Group)
*Lannea acida* (*Odina acida* (A. Rich.) Oliv; *Calesiam acidum* (A.Rich.) Kuntze; *Lannea glaucescens* Engl.; *Lannea lagdoensis* (Engl. & K.Krause) Mildbr.; *Sorindeia lagdoensis* Engl. & K. Krause) [4,13,14]	Benin	Tchemou (ta-kamba), yoronou (bariba), wansawatchemou (waama), zouzou (fon, goum)
	Burkina Faso	Bembé (bambara), ébruhé, ébruké (attié), kondro (baoulé), sambagha, santuluga (mossi), siribu, sisubu (dagari), véké (senoufo)
	Ivory Coast	Béssomo (malinke), sinsàbgà (dagomba)
	Ghana	Gbentore (wale); manvora, vaaworo (lobi)
	Guinea-Bissau	Bembedja, bembem-hei, tchingole (fula); bémbô, irimusso (mandinga); betôlôdje (pepel); dôto (balanta); mantede (criolo); ututene (felupe do Senegal)
	Guinea-Conakry	Bembe nougou (malinké); tiouko, yiouko tioli, thionlli, touko (poular)
	Mali	Bembé (bambara); sìnsàbgà (dagomba); tinyoli (peuhl)
	Niger	Faru, tamarza (zarma)
	Nigeria	Faru (hausa)
	Senegal	Bembô (socé); bembéy (firdou, fouladou); tinoli (peul, tocolor); tuko (peul fouta–djalon)
	Togo	Eberg (gurmantché); gbednatun (moba); kisan, kizan (kabiyé); otchowé (akassélem)
*Lannea alata* (*Calesiam alatum* Engl.; *Lannea minimifolia* (Chiov.) Cufod.; *Odina minimifolia* Chiov.) [15]	Kenya	Borana (wanreh); kumude (bejelo); samburu (mushiga); ngariso tharaka (mituungu)
*Lannea ambacensis* [16]	Angola	Mukumbu kakumbi, mucumbi, mukumbi, mungongolua, ngonjila, umbi
*Lannea angolensis* [17]	Angola	Bulukutu, omunthiwi (kikongo)
*Lannea barteri* (*Calesiam barteri* Kuntze; *Lannea kerstingii* Engl. & K. Krause; *Odina barteri* Oliv; *Lannea kertínger* Engl. & K. Krause.) [18]	Benin	Zuzugoto (fon)
	Camaroon	Sorihi (fulfuldé)
	Guinea-Conakry	Tiuko (aub, fula-fulaar)
	Ivory Coast	Baule kondro, bembe, dinbé, peku (manding-maninka)
	Mali	Bembe, dagaari sisibigolo, sussuguté hausanamisinfara, moore sambituliga, sabagha (aub, begue)
	Nigeria	Báraá as mudas (bargery); tudi (hausa); faru (fulani, hausa)
	Sierra Leone	Dalalonke (susu)
	Togo	Benature, patandĕu, tingbatau (volkens); gurma (manga); met (tshaudjo); aku (yoruba-ife)
*Lannea coromandelica* (*Calesiam grande* (Dennst.) Kuntze, *Dialium coromandelicum* Houtt., *Haberlia grandis* Dennst., *Odina gummifera* Blume; *Odina pinnata* Rottler) [19,20]	Bangladesh	Bhadi, bohar, ghadi, jail, jial bhandi, jiga, jigor, jiol, jir, jival, kasmala, lohar (-)
	India	Annakara, dang paguel-kung, doka, doke, dumpidi, genjan, geru, ginyan, godda, gojal, gumpina, gumpini, jhingan, jingni, Jhingangummi, kalasan, kalayam, kamlai, kashmala, kekat, kiamil, ligna, magir, mohin, moi, mowen, moye, moyen, moyna, nanam, oddi, shimti, udi, uthi, vaddi, oti, ajasrngi (-)
	Myanmar	Maing (-)
	Nepal	Thulo dabdabe (halonre)
	Pakistan	Kembal (-)
*Lannea edulis* (*Lannea nana* Engl; *Odina edulis* Sond; *Calesiam edule* Kuntze.) [20,21]	South Africa	Mutsambatsi (siswati); phepo (setswa-na); umtfokolovu, umgabunkhomo (isizulu); wildedruif (afrikaans)
	Angola	Ngongolua, omungongolua (nyaneka); ngongwila, ungongwila (umubumbu)
	Burundi	Umutabataba (kirundi)
	Kenya	Masungubale (marachi)
	Rwanda	Imbatabata, umutabataba (kinyarwanda)
	Tanza-nia/Uganda	Lihambalimwe (kihehe); makavumba, navakumba (mbozi), mvumvu mkubwa (zaramo, tanzania), nekote (karamojong, ouganda), unahavumba (nyika)
	Zimbabwe	Mutsambatsi (shona)
*Lannea gossweileri* [22]	Angola	African walnut, Gossweileri ash, Gossweileri false ash, Gossweileri Lannea
*Lannea humilis* (*Commiphora taborensis* Engl.; *Lannea bagir*-*mensis* Engl.; *Lannea tomentosa* (Engl.) Engl.; *Odina* humilis Oliv.; *Odina tomentosa* Engl.; *Tapirira humilis* (Oliv.) Marchand.; *Calesiam humile* (Oliv.) Kuntze *Calesiam tomentosum* Engl.) [23]	Nigeria	Kerwúlú, paàruú
	Senegal	Ard a koy, habugan, béluki, ngonaro
	Uganda	Etopojo (ngakarimojong)
*Lannea nigritana* (*Lannea afzelii* Engl.; *Lannea grossularia* A. Chev.; *Odina nigritana* S. Elliot; *Lannea glaberrima* Engl. & K. Krause; *Lannea nigritana* var. *nigritana* Keay; *Odina oghigee* Hook.f.) [4,24]	Guinea-Bissau	Bembedje, bembem-hei, tchingole (fula); bêmbô (mandinga); betôlôdje (pepel); mantede (criolo)
	Guinea-Conakry	Bembé (malinké), lokouré (soussou)
*Lannea rivcae* (*Commiphora tomentosa* Engl; *Lannea cufodontii* Chiov; *Lannea floccosa* Sacleux; *Odina rivae* Chiov.) [20]	Kenya	Kamba, kitharara, kithaala, kithaalua kya kiima, latat, lolowe, marakwet, muthaalwa
*L. schimperi* (*Lannea rufescens* Engl.; *Lannea ruspollii* Engl.; *Lannea schimperi* var. *glabrescens* (Engl.) J.B. Gillett; *Lannea stolzii* Engl. & Brehmer; *Odina schimperi* Hochst. ex A. Rich.); *Calesiam schimperi* (Hochst. ex A.Rich.) Kuntze; *Lannea schimperi* var. *peixe*-*boi*; *Lannea stolzii* Engl. & Brehmer) [25]	Burundi	Igifuto, umufute (kirundi)
	Cameroon	Nkwelegito (babungo)
	Ethiopia	Enxxilif (afaan oromo)
	Mozambique	Munganikomo, xihumbunkany, xivombo nkanyi (changana)
	Namibia	Kangawa (lozi)
	Kenya	Kipng’etingwet, kumubumbu (nandi)
	Sudan	Tony (nuer)
	Tanzania	Mginkinywa (batemi); mugumbu (nyamwezi); navakumba (mbozi); ombumbo (haya)
*Lannea schweinfurthii* (*Calesiam schweinfurthii* (Engl.) Kuntze; *Lannea schweinfurthii* var. *schweinfurthii*; *Odina schweinfurthii* Engl.; *Scassellatia heterophylla* Chiov.) [26]	South Africa	Mi-livhadza (luvenda); mulichadza (venda)
	Mozambique	M’sutototo (chindau)
	Namibia	Rungomba (lozi)
	Kenya	Kuogo (luo); mnyumbu (kilifi); omusalu (suba); mumongoo (pokomo)
	Somalia	Arusha (eravande); gogo (muwumbu); lugu (muhingilo); mate (ndelamwana); mnyamendi, mribwampara, muhondobogo (zinza); msayu, nsayu (suku); mumendo, omosaruwa (kuria); mwera (mpupi); nyam (mnyumbu); pare (msighe); rangi (msakawa); swah (mtundu); tambaragi, thigii (iraqw); zara (mpiwipwi); zigua (mumbu)
	Tanzania	Mbu, mfupapo, mmongo, muumbu, nago (swahili); orpadwa (masaï)
	Uganda	Musinga bakali (bulamogi)
	Zambia	Musamba (silozi)
*Lannea velutina* (*Calesiam velutinum* (A. Rich.) Kuntze; *Odina velutina* Engl. ex Walp.; *Tapirira velutina* Marchand) [4,27]	Benin	(-)
	Burkina Faso	kruntoni (sanan), tougô-dâ, zinzam-tougô (bis-sa), wâamsâbga (mossi)
	Ghana	(-)
	Guinea-Bissau	Aionque (bijagós); ambi-lire (tanda); balêbári (the fruit); bembei, dembei, mantede (criolo); bem-bedje, bembei, bembem- hei, tchucó, tchingole (fula); bémbô (mandinga); be-tôlôdje (pel); coxolourô, cupote-cuxolourô (felupe do senegal); dôtô (balanta); lagari (manjaco); m’riuol (balanta); n’taluass, n’tchalúas, untchalbinass (nalu); n’tata, untata (pepel); sandje-bombo, sand-ji-bombro(fula); undêbári (cobiana)
	Guinea-Conakry	Bembé (malinké), tiouko, tiouko niadouko, tiouko niabé (poular)
	Mali	Bakororonpeku, fégou-ganiè, surukunnpeku (malinke); nteku-bangènyè, bakoro npeku (bambara); satungo npege, saanci jonon (minyanka); satungo vègè (senoufo); sa’ui-nyinu (bwa)
	Niger	(-)
	Senegal	Bemmbeyi (peul), bubu-ka (diola), ndabarndoki (serer), ndogot (wolof), tinolipoley (peul)
	Togo	(-)
*Lannea welwitschii* (*Calesiam welwitschii* Hiern; *Lannea acidíssima* A. Chev.; *Lannea longifoliolata* Engl. & K. Krause; *Lannea zenkeri* Engl. & K. Krause; *Odina welwitschii* K. Schum.; *Ricinodendron staudtii* Pax) [28]	Angola	Nkumbi (kikongo)
	Democratic Republic of the Congo	Kumbi (kikongo)
	Ivory Coast	Loloti, ngdongoloti (abe); kakoro (akanfante); n-nu, nu, tchico, tchiwo (akye); baiséguma, baopiré, bore pore (anyi); trongba (baule); tobero (gagu); tétégné (kru-guere); duko, durgo, duruku (kulango); adubruhia, atukruhia, dugbruhia (kyama); kakoro (nzema)
	Gabon	Okum-nini, kumenini, kum-anini (enti)
	Ghana	Kum-anini, kumenini, kum-onini, kuntunkuni (akan-asante); kakoro (fante); aberewa nyansiŋ, kum-anini, okum-nini (twi); kumenini (wasa); bopire (anyi-sehwi); abalapuli (nzema)
	Nigeria	Abe (loloti); anyisehwi (bopire); anyi (bai-séguma); asante (kuntunkuri); baule (trongba); ekika, ekika-ajá (yoruba); fante (kakoro); gagu (tobero); kulango (duko); kru-guere (tétégné); kyama (adubruhia)

(-)—vernacular name or ethnic group not found.

## Data Availability

Not applicable.
